# Altered expression of cytokines, chemokines, growth factors, and soluble receptors in patients with colorectal cancer, and correlation with treatment outcome

**DOI:** 10.1007/s00262-024-03746-x

**Published:** 2024-07-02

**Authors:** M. Stayoussef, X. Weili, A. Habel, M. Barbirou, S. Bedoui, A. Attia, Y. Omrani, K. Zouari, H. Maghrebi, W. Y. Almawi, B. Bouhaouala-Zahar, A. Larbi, B. Yacoubi-Loueslati

**Affiliations:** 1 Laboratory of Mycology, Faculty of Sciences of Tunis (FST), Pathologies and Biomarkers (LR16ES05), University of Tunis El Manar (UTM), 1092 Tunis, Tunisia; 2 Singapore Immunology Network (SIgN), Agency for Science Technology and Research (A*STAR), Immunos Building, Singapore, 138648 Singapore; 3 Center for Biomedical Informatics, University of Missouri School of Medicine, Columbia, MO USA; 4 Department of Digestive Surgery, Fattouma Bourguiba Hospital, University of Monastir, Monastir, Tunisia; 5 Faculty of Medicine of Tunis, University of Tunis El Manar (UTM), Tunis, Tunisia; 6 Laboratory of Biomolecules, Venoms and Theranostic Applications, University of Tunis El Manar (UTM), Pasteur Institute of Tunis, 13 Place Pasteur, B.P. 74, 1002 Tunis, Tunisia; 7 University of Tunis El Manar (UTM), Medical School of Tunis, Rue Djebal Lakhdar, 1006 Tunis, Tunisia; 8 Department of Medicine, Faculty of Medicine and Health Sciences, University of Sherbrooke, Sherbrooke, QC Canada

**Keywords:** Biomarker discovery, Chemokines, Chemoresistance, Colorectal cancer, Cytokines, Metastasis

## Abstract

Insofar as they play an important role in the pathogenesis of colorectal cancer (CRC), this study analyzes the serum profile of cytokines, chemokines, growth factors, and soluble receptors in patients with CRC and cancer-free controls as possible CRC signatures. Serum levels of 65 analytes were measured in patients with CRC and age- and sex-matched cancer-free controls using the ProcartaPlex Human Immune Monitoring 65-Plex Panel. Of the 65 tested analytes, 8 cytokines (CSF-3, IFN-γ, IL-12p70, IL-18, IL-20, MIF, TNF-α and TSLP), 8 chemokines (fractalkine, MIP-1β, BLC, Eotaxin-1, Eotaxin-2, IP-10, MIP-1a, MIP-3a), 2 growth factors (FGF-2, MMP-1), and 4 soluble receptors (APRIL, CD30, TNFRII, and TWEAK), were differentially expressed in CRC. ROC analysis confirmed the high association of TNF-α, BLC, Eotaxin-1, APRIL, and Tweak with AUC > 0.70, suggesting theranostic application. The expression of IFN-γ, IL-18, MIF, BLC, Eotaxin-1, Eotaxin-2, IP-10, and MMP1 was lower in metastatic compared to non-metastatic CRC; only AUC of MIF and MIP-1β were > 0.7. Moreover, MDC, IL-7, MIF, IL-21, and TNF-α are positively associated with tolerance to CRC chemotherapy (CT) (AUC > 0.7), whereas IL-31, Fractalkine, Eotaxin-1, and Eotaxin-2 were positively associated with resistance to CT. TNF-α, BLC, Eotaxin-1, APRIL, and Tweak may be used as first-line early detection of CRC. The variable levels of MIF and MIP-1β between metastatic and non-metastatic cases assign prognostic nature to these factors in CRC progression. Regarding tolerance to CT, MDC, IL-7, MIF, IL-21, and TNF-α are key when down-regulated or resistant to treatment is observed.

## Introduction

Colorectal cancer (CRC) is the third most prevalent cancer type, and the fourth most deadly cancer worldwide, with an estimated 916,000 annual deaths [1]. The prevalence of CRC in Tunisia has increased in recent years from 6.4/100,000 in 1994 to 12.4/100,000 in 2009 and further projected increases to 39.3/100,000 in 2024 were reported [2]. The pathogenesis of CRC depends on the presence of modifiable and non-modifiable factors, the latter include mutations, microsatellite status, methylation alterations, and tumor microenvironment [3–5]. In this regard, the progression from pre-cancerous polyps to cancerous tumors in the colon or rectum is a multistep process that spans more than 15 years, thereby providing an opportunity for early detection, and likely prevention, by the removal of premalignant polyps [6].

Several methods are available for CRC screening and are categorized into invasive and non-invasive tests. The former includes the fecal occult blood test, the fecal-based DNA test, and the fecal immunochemical test [7]. On the other hand, invasive tests include colonoscopy and flexible sigmoidoscopy and are sensitive and specific but cannot be applied for everyone in light of the heightened risk of complications, and the high costs [7, 8]. The shortcomings associated with these and other CRC screening methods, coupled with lifestyle, immunogenetic and related factors [9] negatively impacted their ability for early-stage/new-onset disease detection, necessitating the search for alternative, minimally invasive robust detection methods.

Soluble proteins such as cytokines, chemokines, growth factors, and receptors are small proteins that show robust modulation and regulation during the immune response and repair mechanisms during which inflammation is key [3, 4, 8]. Chronic inflammation is central to the progression of, and survival in CRC, since it contributes to tumor angiogenesis, invasion and metastasis [10]. Cytokine secretion promoted by chronic inflammation during stages of cancer reportedly induces DNA damage via increased reactive oxygen and nitrogen species, as well as the epigenome of the cells [11–13]. The chemokine networks comprising chemokines and chemokine receptors also contribute to the development and progression of CRC, highlighted by the finding that binding of chemokines to corresponding receptors on vascular endothelial cells modulate tumor development, angiogenesis, invasion, and metastasis [14]. The complex interactions of aberrantly expressed cytokines, chemokines, growth factors, and matrix-remodeling enzymes promote CRC pathogenesis and evoke systemic responses that affect disease outcomes. In addition, these soluble proteins may serve as potential biomarkers, tools for screening, and diagnosis classification between stages of diseases or surveillance following therapy [5, 15]. To this end, assessment of soluble protein levels may be useful for screening, diagnosis classification, and/or monitoring following therapy.

To date, diagnostic and prognostic tools available to early detect and estimate the survival of patients with CRC are limited. This study was designed to compare the profile of key inflammatory cytokines, chemokines, receptors and growth factors in CRC and healthy controls. These 65 soluble immune mediators were investigated in the serum of CRC patients based on their metastatic status and response to CT, in order to identify potentially new theranostic biomarkers that can be utilized in the context of CRC.

## Subjects and methods

### Study subjects

This retrospective cross-sectional study was conducted in the Oncology Service of Salah Azaiez Hospital (Tunis, Tunisia) using a protocol consistent with the Declaration of Helsinki II guidelines and approved by the research and ethics committee of Salah Azaiez Hospital, and informed signed consent was obtained from all participants. Between February 2018 and October 2019, eighty-eight patients with CRC diagnosis (37 males, 51 females) (mean age, 59.4 ± 12.7 years) were recruited from the Oncology Service of Salah Azaiez Hospital (Tunis, Tunisia). CRC assessment was based on clinical examination, colonoscopy, and histopathological findings. Pathological and clinical parameters were obtained from medical records, while demographic information was collected by a personal unified questionnaire. These included gender, age, and body mass index (BMI) at study entry, along with CRC diagnosis, history of anemia, hypertension, cancer (personal and family), and smoking (never, current, and ex-smoker). Histological evaluation, tumor location (rectum or colon/sigmoid), and CT treatment were recorded for all patients. Excluded from the study were patients who developed CRC aged over 40 years at diagnosis and those with a strong family history of CRC or other relevant cancers.

CRC patients were divided based on their metastatic status and response to CT, in order to identify potentially new theranostic biomarkers that can be utilized in the context of CRC. CRC chemotherapy (CT) consisted of cytotoxic drugs administered either as an injection or orally [10]. Intravenous FOLFOX, a combination of FOL (folinic acid and Leucovorin calcium), F fluorouracil (5-FU, Adrucil), LV5FU2 and OX (oxaliplatin, Eloxatin), is the most commonly used adjuvant therapy in CRC. In case of non-tolerance to FOLFOX, oral Xeloda-type CT is given. For metastatic CRC, FOLFOX, FOLFIRI, LV5FU2, or Capecitabine, were used. Intolerance to CT was based on toxicity, discontinuation of therapy, dose reduction, functional decline, and mortality during or shortly following commencing CT.

Control subjects comprised 78 age-, gender-, and BMI-matched healthy university and hospital employees, or volunteer subjects (mean age 57.4 ± 13.3 years). None of control subjects reported personal or family history of CRC, and were matched to CRC cases according to self-declared ethnic origin.

### Blood sampling

Five milliliters of venous blood were collected in a sterile serum tube with no anti-coagulants. Following centrifugation within 2 h after collection (2,500 rpm for 20 min), serum samples were transferred into a micro-centrifuge tube, followed by a second-high speed centrifuge (14,000 rpm, 10 min) to remove cellular debris, were aliquoted and were stored in small aliquots at − 80 °C pending use.

### Multiplex immunoassay

The Immune Monitoring 65-Plex Human ProcartaPlex™ Panel (ThermoFisher Scientific; Nimes, France) was applied on the Bio-Plex 200 suspension array system (Bio-Rad Laboratories, Hercules, CA) for measuring the serum levels of 65 targeted proteins (ng/ml). These included 33 cytokines (G-CSF, GM-CSF, IFN-α, IFN-γ, IL-1α, IL-1β, IL-2, IL-3, IL-4, IL-5, IL-6, IL-7, IL-8, IL-9, IL-10, IL-12p70, IL-13, IL-15, IL-16, IL-17A, IL-18, IL-20, IL-21, IL-22, IL-23, IL-27, IL-31, LIF, M-CSF, MIF, TNF-α, TNF-β, and TSLP), 18 chemokines (BLC, ENA-78, Eotaxin-1, Eotaxin2, Eotaxin-3, Fractalkine, GROα, IP-10, I-TAC, MCP-1, MCP-2, MCP-3, MDC, MIG, MIP-1α, MIP-1β, MIP-3α, and SDF-1α), 6 growth factors/regulators (FGF-2, HGF, MMP-1, NGF-β, SCF, VEGF-A), and 8 soluble receptors (APRIL, BAFF, CD30, CD40L, IL-2R, TNF-RII, TRAIL, and TWEAK), which were tested as 50 ul samples according to the instructions of the manufacturer. Detailed assay protocol can be accessed at: https://www.thermofisher.com/order/catalog/product/EPX650-10065-901

### Statistical analysis

Basic statistical analysis was done using SPSS 24.0 (IBM, Armonk, NY), and the graphs were generated using GraphPad Prism 7.0 (GraphPad Software, San Diego, CA). Continuous data were expressed as the mean ± SD, and inter-group differences between CRC patients and healthy controls, and among CRC patients stratified according to their response to the treatment (sensitive or resistant), tolerance to treatment (tolerant or non-tolerant) were performed using Mann–Whitney-test. Receiver operating characteristics (ROC) analysis was performed using SPSS; the Area-Under-ROC- Curves (AUC) ± SD, and *P* values were calculated for all tested analytes; *P* < 0.05 was considered statistically significant.

## Results

### Demographic and clinical characteristics

The demographic and clinical profile of the 88 patients with CRC, and 78 cancer-free healthy subjects; all being Tunisians, is presented in Table 1. The average age (59.4 ± 12.7 and 57.4 ± 13.3 years; *P* = 0.32), mean BMI (25.1 ± 4.1 and 26.8 ± 6.3 kg/m^2^; *P* = 0.13); alcohol consumption (16.1% and 25.6%; *P* = 0.14) and smoking (24.4% and 27.2%; *P* = 0.69) were comparable between patients with CRC and cancer-free control subjects. While 46.9% of all CRC patients presented with metastasis, the exact location of the site of the metastasis (liver, lymph nodes, etc.) were not established for all patients. This was due to patient (terminal cases, personal/family consideration), and technical considerations. Patients were stratified according to resistance to CT into treatment-resistant (R + ; 29.6%), and treatment-sensitive (R − , 45.7%), as well as treatment-tolerant (T + , 11.1%), and treatment-nontolerant (T − , 60.5%)].

**Table 1:** Demographic and clinical characteristics of study participants

Characteristic	CRC Cases (n = 88)	Controls (n = 78)	*P *^*a*^
Gender (Males/Females)^b^	37:51 (45.7:63)	29:49 (37.2: 62.9)	0.522
Age (yr)^c^	59.4 ± 12.7	57.4 ± 13.3	0.322
BMI (kg/m^2^) ^3^	25.1 ± 4.1	26.8 ± 6.3	0.128
Smoking^b^	22 (24.4)	21 (27.2)	0.686
Alcohol^b^	13 (16.1)	20 (25.6)	0.136
Metastasis^b^ M- M + Unknown	41 (50.61) 38 (46.91) 2 (2.46)	NA	–
CT responsive^b^ R- R + Unknown	24 (29.62) 37 (45.67) 20 (24.69)	NA	–
CT tolerant ^2^ T- T + Unknown	9 (11.11) 49 (60.5) 23 (28.39)	NA	–

CRC, colorectal cancer; CT, chemotherapy; HC, healthy controls; M-, non-metastatic patients. M + , metastatic; R + : treatment-sensitive; R-, treatment-resistant; T-, tolerant to treatment; T + , non-tolerant to treatment; NA, not applicable

^a^Pearson chi-square (categorical variables), Student t-test (continuous variables)

^b^Number (percent total)

^c^Mean ± SD

### Association of the analytes with the development of CRC

**Fig. 1:**
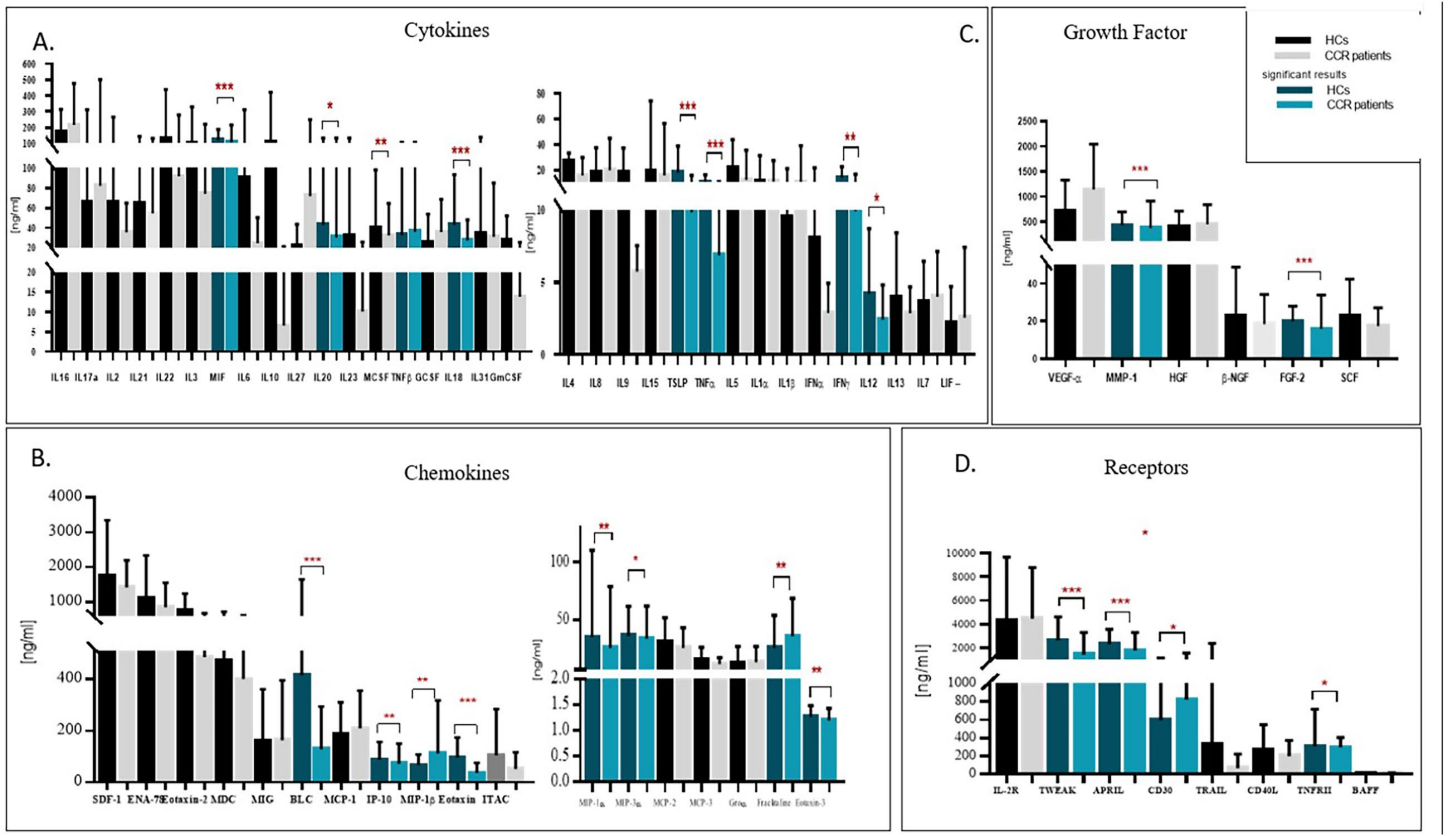
Serum levels of tested analytes in CRC cases and healthy controls. **A** Serum cytokine levels (left, cytokines with mean levels > 5 ng/ml; right: cytokines with mean level < 5 ng/ml); **B** Serum chemokine levels (left, chemokines with mean level > 40 ng/ml; right, chemokines with mean level < 40 ng/ml); **C** Serum growth factor levels in CRC and **D** Serum soluble receptor levels in CRC cases and healthy controls. *** *P* < 0.0001; **P < 0.005; *P < 0.05

Twenty-two analytes comprising 8 cytokines, 8 chemokines, 2 growth factors, and 4 soluble receptors, were differentially expressed between CRC and healthy controls (Table 2). Of the eight cytokines, CSF-3 (*P* = 4.0 × 10^–4^) was downregulated, while IFN-γ (*P* = 2.0 × 10^–4^), IL-12p70 (*P* = 0.016), IL-18 (*P* < 0.001), IL-20 (*P* = 0.012), MIF (*P* = 7.0 × 10^–3^), TNF-α (*P* < 0.001), and TSLP (*P* < 0.001) were upregulated in the sera of patients with CRC compared to healthy controls (Table 2, Fig. 1A). Of the 8 differentially-expressed chemokines, serum levels of Fractalkine (*P* = 3.0 × 10^–4^) and MIP-1β (*P* = 4.6 × 10^–3^) were significantly lower, while those of BLC (*P* < 0.001), Eotaxin-1 (*P* < 0.001), Eotaxin-2 (*P* = 6.6 × 10^–3^), IP-10 (*P* = 6.9 × 10^–3^), MIP-1a (*P* = 5.4 × 10^–3^) and MIP-3a (*P* = 0.032) were significantly higher in the sera of patients with CRC compared with those of healthy controls (Table 2, Fig. 1B).

**Table 2:** Serum proteins that are differentially expressed between CRC patients and healthy controls

		ANALYTES ^1^	CRC Cases ^2^	Controls ^2^	P value ^3^	AUC
Cytokines	Down-regulated	CSF-3	25.3 ± 28.4	35.4 ± 33.2	4.0 × 10^–4^	0.671
	Up-regulated	IFN.γ	14.0 ± 8.6	9.9 ± 6.8	2.0 × 10^–4^	0.676
		IL.12p70	4.1 ± 4.4	2.4 ± 2.4	0.016	0.637
		IL-18	43.0 ± 50.4	27.3 ± 20.7	< 1.0 × 10^–4^	0.695
		IL-20	42.9 ± 91.0	30.3 ± 102.9	0.012	0.636
		MIF	120.3 ± 69.0	106.9 ± 108.7	7.3 × 10^–3^	0.627
		TNF-α	10.4 ± 5.9	6.9 ± 3.9	< 1.0 × 10^–4^	0.704
		TSLP	18.2 ± 20.5	9.8 ± 6.0	< 1.0 × 10^–4^	0.689
Chemokines	Down-regulated	Fractalkine	25.3 ± 28.4	35.4 ± 33.2	3.0 × 10^–4^	0.671
		MIP-1β	67.0 ± 41.0	111.4 ± 206.3	4.6 × 10^–3^	0.634
	Up-regulated	BLC	414.3 ± 123.0	128.4 ± 165.1	< 1.0 × 10^–4^	0.700
		Eotaxin-1	96.1 ± 77.28	36.8 ± 39.3	< 1.0 × 10^–4^	0.803
		Eotaxin-2	739.8 ± 499.0	484.5 ± 184.6	6.6 × 10^–3^	0.629
		IP.10	87.5 ± 69.4	71.7 ± 78.7	6.9 × 10^–3^	0.628
		MIP-1a	36.13 ± 76.6	25.3 ± 53.6	5.4 × 10^–3^	0.634
		MIP-3a	35.6 ± 25.9	33.2 ± 28.8	0.032	0.602
Growth-Factors	Up-regulated	FGF-2	19.8 ± 8.3	16.0 ± 18.0	< 1.0 × 10^–4^	0.690
		MMP-1	422.0 ± 270.8	366.5 ± 545.3	2.0 × 10^–4^	0.674
Soluble Receptors	Down-regulated	APRIL	525.1 ± 143.0	557.8 ± 102.9	< 1.0 × 10^–4^	0.710
		CD30	598.1 ± 545.1	825.9 ± 728.9	0.015	0.615
	Up-regulated	TNF-RII	306.7 ± 410.3	295.8 ± 110.2	0.035	0.600
		Tweak	262.4 ± 198.9	141.3 ± 187.4	< 1.0 × 10^–4^	0.760

AUC, Area Under the Curve

1.ng/ml

2.Mean ± SD

3. Determined by Mann–Whitney test

Among the tested growth factors, FGF-2 (*P* < 0.0001) and MMP-1 (*P* = 0.0054) were the only factors that were upregulated in the sera of patients with CRC compared to healthy controls (Table 2, Fig. 1C). Of the soluble-form receptors included, APRIL (*P* < 0.0001) and CD30 (*P* = 0.015) were significantly reduced, in contrast to TNF-RII (*P* = 0.035) and Tweak (*P* < 0.0001) which were over-expressed in patients with CRC than in healthy controls (Table 2, Fig. 1D). ROC curves confirmed the altered association of these 21 factors with altered risk of CRC, suggesting a potential discriminative potential for CRC detection (AUC > 0.6), with TNF-α, BLC, Eotaxin-1, APRIL, and Tweak having a likely potential clinical utility, as verified by the relatively high (> 0.70) AUC values (Table 2).

### Association with metastasis and resistance to therapy

**Fig. 2:**
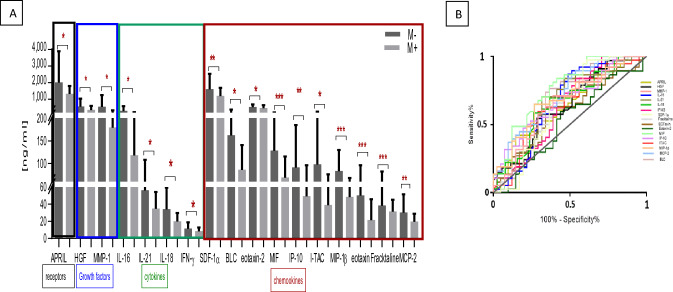
**A** Analytes significantly differentially expressed in metastatic versus non metastatic patients with CRC. **B** ROC curves for the analytes significantly expressed differentially between metastatic and non-metastatic CRC patients. Results are expressed as median values (ng/ml); *P < 0.05

We tested the notion that differential serum biomarker expression is linked with altered CRC metastatic capacity and/or tolerance to CT by stratifying patients with CRC into metastatic vs. non-metastatic, and CT-resistant vs. CT-sensitive groups. Results from Fig. 2A and Table 3 showed that the serum levels of APRIL (*P* = 0.019), the growth factors HGF (*P* = 0.036) and MMP-1 (*P* < 0.001)], as well as the cytokines IL-16 (*P* = 0.041), IL-21 (*P* = 0.018), IL-18 (*P* = 0.011), and IFN-γ (*P* = 0.030), and the chemokines SDF-1α (*P* = 0.004), BLC (*P* = 0.038), Eotaxin-2 (*P* = 0.046), MIF (*P* = 7.0 × 10^–4^), IP-10 (*P* = 9.0 × 10^–3^), I-TAC (*P* = 0.046), MIP-1β (*P* < 0.001), Eotaxin-1 (*P* < 0.001), Fractalkine (*P* < 0.001) and MCP-2 (*P* = 6.4 × 10^–3^)] were significantly lower in patients with CRC who developed distant metastases. The majority of these factors had acceptable AUCs, with MIF (0.71) and MIP-1β (0.75) presenting with high AUC; suggesting a potential prognostic utility of these chemokines in patients who might develop metastasis (Fig. 2B) (Table 3).

**Table 3:** Serum proteins differentially expressed between CRC non-metastatic and metastatic patients

	ANALYTES^a^	Non-metastatic	Metastatic	*P* value^a^	AUC
Cytokines	IL-16	286.5 ± 300.8	117.6 ± 107.6	0.041	0.63
	IL-21	56.1 ± 51.7	34.62 ± 19.9	0.018	0.65
	IL-18	34.1 ± 25.4	19.85 ± 10.12	0.011	0.66
	IFN-γ	11.4 ± 7.7	8.195 ± 4.799	0.030	0.64
Chemokines	SDF-1α	327.5 ± 827.9	1192.0 ± 494.9	4.3 × 10^–3^	0.68
	BLC	161.9 ± 214.4	85.7 ± 55.1	0.038	0.63
	Eotaxin-2	2.8 ± 0.3	2.6 ± 0.3	0.046	0.58
	MIF	128.0 ± 85.8	68.3 ± 46.9	7.0 × 10^–4^	0.71
	IP-10	90.8 ± 94.2	49.2 ± 46.7	9.0 × 10^–3^	0.67
	I-TAC	97.6 ± 158.4	39.1 ± 36.8	0.046	0.64
	MIP-1β	82.6 ± 47.3	48.2 ± 19.65	< 1.0 × 10^–4^	0.75
	Eotaxin	1.8 ± 0.4	1.1 ± 0.6	< 1.0 × 10^–4^	0.67
	Fractalkine^*^	1.3 ± 0.4	1.5 ± 0.2	< 1.0 × 10^–4^	0.52
	MCP-2	30.0 ± 21.8	19.6 ± 9.4	6.4 × 10^–3^	0.67
Growth-Factors	HGF	553.0 ± 476.7	353.5 ± 241.8	0.036	0.63
	MMP-1	539.5 ± 716.8	179.2 ± 153.4	< 1.0 × 10^–4^	0.63
Soluble Receptors	APRIL	2,001.0 ± 1,888.0	1,320 ± 481.5	0.019	0.65

AUC, Area Under the Curve

^a^ ng/ml

^b^*P* value performed by Mann–Whitney test. 2:

**Fig. 3:**
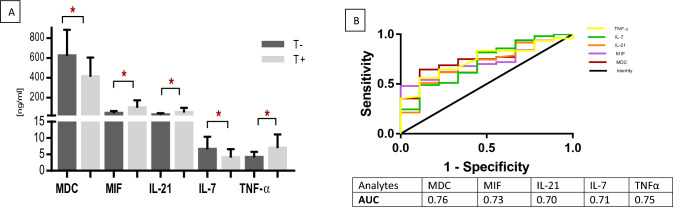
**A** Analytes significantly expressed differentially in chemotherapy-tolerant and -non tolerant patients with CRC. **B** ROC curves for the five analytes significantly expressed differentially between chemotherapy-tolerant and -nontolerant patients with CRC. *** *P* < 0.0001; **P < 0.005; *P < 0.05

**Fig. 4:**
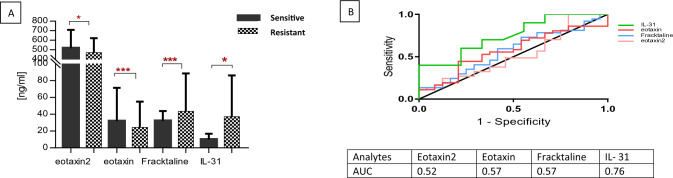
**A** Analytes expressed between chemotherapy-sensitive and -resistant patients with CRC. **B** ROC curves for the five analytes expressed in chemotherapy-sensitive and -resistant patients with CRC. *** *P* < 0.0001; **P < 0.005; *P < 0.05

Based on their tolerance to CT, the cytokines MIF (*P* = 0.033), IL-21 (*P* = 0.050), IL-7 (*P* = 0.039), and TNF-α (*P* = 0.0206), and the chemokine MDC (*P* = 0.013) were positively associated with CT tolerance in patients with CRC (Fig. 3A). The high AUC found for MDC (0.76), MIF (0.73), IL-21 (0.70), IL-7 (0.71), and TNF-α (0.75) represented novel biomarkers for identifying tolerance to CT (Fig. 3B). In addition, the chemokines Eotaxin-2 (*P* = 0.046), Eotaxin-1 (*P* < 0.001), and Fracktaline (*P* < 0.001), and the cytokine IL-31 (*P* = 0.046) were positively associated with resistance to CRC CT (Fig. 4A). While the identified chemokines had acceptable AUC, IL-31 cytokine had a high AUC (0.76), and thus a potential diagnostic value. Collectively, this highlights the potent theranostic application of these factors in identifying patients with CRC who may develop resistance to treatments (Fig. 4B).

## Discussion

Using a discovery approach, we tested the diagnostic and prognostic capacity of altered expression of multiple soluble factors in CRC. We documented up-regulation in the expression of IFN-γ, IL12p70, IL-18, IL-20, MIF, TNF-α, TSLP, BLC, Eotaxin-1, Eotaxin-2, IP-10, MIP-1a, MIP-3a, FGF-2, MMP-1, TNFRII and TWEAK, which are implicated in the modulation of inflammation, apoptosis, tumor growth, invasion, and angiogenesis. This is in accord with earlier studies documenting the increased levels of these and related proinflammatory factors in human malignancies [16, 17] including CRC [3].

The elevation in serum levels of the identified mediators in patients with CRC patients underscores the state of inflammation accompanying CRC [18, 19]. It was shown that heightened IFN-γ levels are generally tumor-suppressive [8, 19], and that deficiency of IFN-γ or its receptor-promoted colorectal carcinogenesis [20], thus prompting the conclusion of a key role for IFN-γ in patient survival [3, 21]. Insofar as IL-12p70 stimulates IFN-γ production, a diagnostic/prognostic role of increased IL12p70 levels in CRC was suggested [22, 23]. An anti-tumor role for IL-18 (formerly called IFNγ-inducing factor) was also proposed, based on its capacity to stimulate the production of IFN-γ by activated lymphocytes, subsequently enhancing apoptosis of tumor cells [24]. On the other hand, IFN-γ-induced protein 10 (IP-10), induced by IFN-γ and up-regulated in response to inflammatory cytokines, including TNF-α [20], was shown to act in an autocrine fashion to improve tumor cell proliferation, angiogenesis, and metastasis [25].

IFN-γ and TNF-α are key in the inflammatory process associated with CRC [8, 26] and reportedly act by inducing activation of NF-κB [26, 27]. Overexpression of TNF-related weak inducer of apoptosis (Tweak) was noted in the present study, in agreement with earlier reports [28], acting by regulating TNF activity and attenuating the transition from innate to adaptive immunity. Furthermore, heightened expression and signaling of the TNF-α receptor, TNFR2, promotes tumor growth, as also shown elsewhere [29].

Our results are in accordance with previous studies indicating that a higher level of circulating MMP-1 is related to prognosis in patients with colon cancer [30, 31]. Similar to our findings, FGF2 gene expression was shown in recent reports to be elevated in colorectal tumor tissue [32]. Furthermore, the increased expression of B-lymphocyte chemoattractant (BLC) reported here is consistent with earlier studies on its role in CRC pathogenesis, since deficiency and the blockade of BLC signaling attenuates disease progression [33]. Functionally, BLC was shown to promote intestinal tumorigenesis through the activation of the AKT signaling pathway [29]. Insofar as eotaxin-1 (CCL-11) is an eosinophil chemoattractant, and as tumor-associated tissue eosinophilia is linked with improved CRC prognosis [34], heightened Eotaxin-1 in CRC was confirmed here and elsewhere [8, 35]. In addition to Eotaxin-1, Eotaxin-2 constitutes a likely mechanism against the destruction of CRC cells, and high Eotaxin-2 plasma levels were associated with CRC-specific mortality [36].

The macrophage Inflammatory proteins (MIP) MIP-1a (/CCL3) and MIP-3a (CCL20) are chemotactic chemokines that can promote the proliferation of CRC cells [8, 35]. The elevated MIP-1α plasma levels in patients with CRC compared with healthy controls reported here and elsewhere [8], coupled with the reported upregulation of MIP-3a in colorectal adenomas, colorectal adenocarcinomas, and colorectal liver metastases tissues [37], and its correlation with poor prognosis of CRC [37, 38], highlights the key role MIPs play in the pathogenesis of cancers, including CRC.

On the other hand, reduced expression of CSF-3, Fractalkine, MIP-1β, APRIL, and CD30 was seen in patients with CRC. This was the first study to investigate the association of altered CSF3 serum levels with the risk of CRC, and additional studies from different ethnic groups are warranted for confirming the association of CSF3 as a theranostic biomarker with CRC. Our results showed that Fractalkine/CX3CL1 was downregulated in patients with CRC compared to control subjects, suggesting a favorable prognosis. This was in agreement with earlier reports documenting the association of increased intra-tumor expression of Fractalkine/CX3CL1 with a favorable prognosis in CRC [39, 40]. In addition, lower levels of MIP-1β in patients with CRC stage IV compared to healthy controls were noted, which was reminiscent of the recent findings on human CRC cell lines, and also an animal model for CRC liver metastasis [41], in which MIP-1β/CCL4 was implicated in carcinogenesis, likely by facilitating instability in the tumor environment [42]. Noteworthy here was that almost half of the included patients were at stage IV of CRC.

The downregulation of proliferation-inducing ligand (APRIL) in patients with CRC compared to control subjects, was consistent with its role in regulating the survival and proliferation of cancer cells [43]. Our results were in apparent disagreement with an earlier Dutch study, which reported on heightened levels of APRIL in CRC before surgery, and poor prognosis dependent on the stage of the disease [44]. These differences are reconciled by differences in disease states, treatment modalities, and likely genetic background of study subjects. We also documented decreased CD30 serum levels in CRC patients than in healthy controls, in agreement with a recent report documenting its association with a favorable outcome [45]. Collectively, this assigns a diagnostic and prognostic aspect to the measurement of these factors.

Functionally, lower levels of IFN-γ, IL-18, MIF, BLC, Eotaxin-1, Eotaxin 2, IP-10, and MMP1 were seen in metastatic compared to non-metastatic CRC cases, suggesting a protective nature of these factors in CRC metastasis. This was in agreement with a Kazakhstani study that reported on reduced MIF serum levels in metastatic CRC [46], and a Taiwanese study that documented that BLC deficiency and blockade of signaling attenuate CRC progression and metastasis [33]. Our study is the first to describe the favorable outcome of Eotaxin-1 and Eotaxin-2 on CRC metastasis. Lower levels of APRIL, MIP-1β, and Fractalkine in metastatic CRC cases compared to both healthy controls and non-metastatic CRC cases. consistent with the association of APRIL silencing with reduced tumorigenesis and metastasis of CRC cells [47, 48]. Consistent with our findings, recent studies demonstrated lower concentrations of MIP-1β in CRC stage IV, and a role of the Fractalkine-receptor axis as a retention factor was suggested. Accordingly, this increases homotypic cell adhesion and improves CRC prognosis by limiting tumor metastatic dissemination [39, 40].

In addition, we demonstrated reduced expression of HGF, IL-16, IL-21, SDF-1α, I-TAC, and MCP2 in metastatic as compared to non-metastatic CRC cases. HGF and its receptor, c-Met, promote tumorigenesis by regulating proliferation, motility, invasion, and metastasis [49], including CRC [50]. The reduction in IL-21 expression was reminiscent of an earlier study, which documented that IL-21 deficiency correlates with an enhancement of the Th17 axis in sporadic intestinal tumor genesis [51]. On the other hand, the reduction in SDF-1α in metastatic CRC is likely attributed to the SDF-1α-CXCR4 interaction and thus promotion of epithelial to the mesenchymal transmission of colon cancer cells, and hence metastasis [52]. In contrast, the interaction of SDF-1α with the alternate CXCR7 stimulates ERK and Akt pathways and enhances angiogenesis [52]. While induced by IFN-γ [52, 53], low serum I-TAC levels seen in metastatic CRC cases may be explained by downstream CXCL11secretion, which produces opposite effects on tumor proliferation and metastases depending on the interacting ligands (CXCR3, CXCR7) [25, 53]. Lastly, the downregulation of MCP-2 in metastatic CRC is attributed to its cellular source (NK cells, fibroblasts, or macrophages), and thus its activity in controlling metastasis [54, 55].

Reduction in MDC and IL-7 coupled with increases in MIF, IL-21, and TNF-α serum levels were found in patients with CRC who are tolerant to CT, as confirmed with the good sensitivity and specificity on ROC analysis. While not fully examined here, these soluble factors control CT resistance by engaging downstream survival-related signaling pathways [56, 57]. On the other hand, increased serum levels of Fractalkine (CX3CL1) and IL-31, along with Eotaxin-1 and Eotaxin-2, were seen in patients resistant to CT. While the exact mechanisms underlying the association between the circulating cytokine levels and the chemoresistance in CRC are not fully understood, it is possible that it may involve upregulating the expression of other proinflammatory cytokines and chemokines, including IL-6 [58]. In turn, this precipitates CT resistance in CRC cells, as was demonstrated in eosinophils and dendritic cells [59]. It is tempting to speculate that this may allow the development of simple and affordable theranostic tools for predicting responsiveness for CT in patients with CRC.

In conclusion, we identified four biomarker panels (22 analytes) to be strongly associated with CRC and thus have CRC diagnostic potential. We also defined 17 analytes to be downregulated in metastatic patients and two panels associated with tolerance (five analytes) and resistance (four analytes) to CT. Given its retrospective design, we could not perform a survival analysis associated with the identified markers. These are of particular therapeutic significance in identifying resistance to CT. Further studies are needed to confirm the role of cytokines, chemokines, growth factors, and soluble receptors in the mechanisms of tolerance or resistance to CT in CRC.

### Clinical practice points

Previous studies established the key role cytokines, chemokines, growth factors, and soluble receptors play in the pathogenesis of colorectal cancer (CRC). The new findings of this study were the association of altered expression of eight cytokines, eight chemokines, two growth factors, and further four soluble receptors with altered risk of CRC, of which TNF-α, BLC, Eotaxin-1, APRIL, and Tweak were highly significant. Altered expression of IFN-γ, IL-18, MIF, BLC, Eotaxin-1, Eotaxin-2, IP-10, and MMP1 was noted in metastatic CRC, while MDC, IL-7, MIF, IL-21, and TNF-α were associated with CT tolerance, while whereas IL-31, Fractalkine, Eotaxin-1, and Eotaxin-2 were linked with resistance to treatment. The altered expression of TNF-α, BLC, Eotaxin-1, APRIL, and Tweak can serve an early first-line diagnostic and prognostic role in CRC detection. On the other hand, variation in the levels of MIF and MIP-1β in metastatic CRC assign prognostic nature to these factors, while the association of MDC, IL-7, MIF, IL-21, and TNF-α with tolerance to therapy can be instrumental towards tailored therapy of CRC.

## Data Availability

Datasets generated during and/or analyzed during the current study are available from the corresponding author on reasonable request.”
